# Etymologia: *Taenia saginata*

**DOI:** 10.3201/eid2312.ET2312

**Published:** 2017-12

**Authors:** Ronnie Henry

**Keywords:** Taenia saginata, tapeworm, beef tapeworm, cestode, parasites, zoonoses, Johann Goeze

## *Taenia saginata* [teʹne-ə sajʺe-naʹta]

Johann Goeze is credited with the first correct description of *Taenia* [Latin, “flat band” or “ribbon”] *saginata* [Latin, “fed”], commonly known as the beef tapeworm, in 1782. Historically, *Taenia* tapeworms (Figure) were believed to have infected humans no more than 10,000 years ago, around the time of domestication of cows and pigs.

**Figure:**
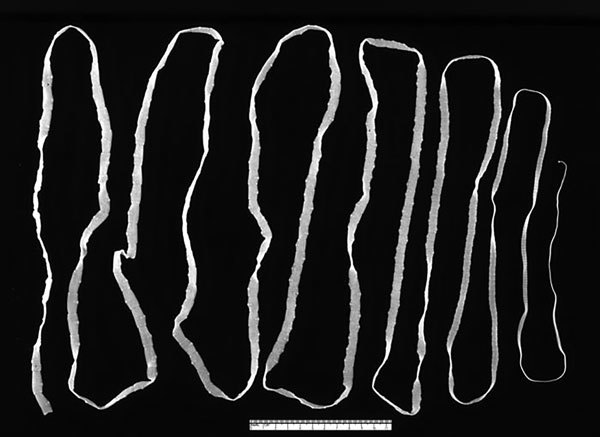
Ad *Taenia saginata* tapeworm. Photo CDC/1986.

However, more recent phylogenetic evidence suggests that ancestors of modern humans, living on the savannahs of Africa and preying on antelope and other bovids, became colonized with *Taenia* >3 million years ago. Parasite definitive hosts switched from large carnivores (probably hyenas) to hominids through their common prey, and this process triggered the evolution of human-infecting species of *Taenia.* Humans later spread these parasites to domestic animals.
